# Applications of Microbial β-Mannanases

**DOI:** 10.3389/fbioe.2020.598630

**Published:** 2020-12-15

**Authors:** Aneesa Dawood, Kesen Ma

**Affiliations:** ^1^Department of Microbiology, Quaid-I-Azam University, Islamabad, Pakistan; ^2^Department of Radiology and Medical Imaging, University of Virginia, Charlottesville, VA, United States; ^3^Department of Biology, University of Waterloo, Waterloo, ON, Canada

**Keywords:** hemicellulose, microbial β-mannanase, industrial applications, bioengineering, heterologous production

## Abstract

Mannans are main components of hemicellulosic fraction of softwoods and they are present widely in plant tissues. β-mannanases are the major mannan-degrading enzymes and are produced by different plants, animals, actinomycetes, fungi, and bacteria. These enzymes can function under conditions of wide range of pH and temperature. Applications of β-mannanases have therefore, been found in different industries such as animal feed, food, biorefinery, textile, detergent, and paper and pulp. This review summarizes the most recent studies reported on potential applications of β-mannanases and bioengineering of β-mannanases to modify and optimize their key catalytic properties to cater to growing demands of commercial sectors.

## Introduction

Hemicellulose is the second most abundant polymer found in nature. It is usually present together with lignin and cellulose in the plant cell walls (Harris and Stone, 2009). It is estimated that hemicellulose is a third of the total components in plants, for example, hemicellulose makes up 25–30% of the total weight of dry wood. Hetero-1,4-β-D-mannans and hetero-1,4-β-D-xylans are two most significant types of hemicelluloses (Chauhan et al., 2012).

**FIGURE 1:**
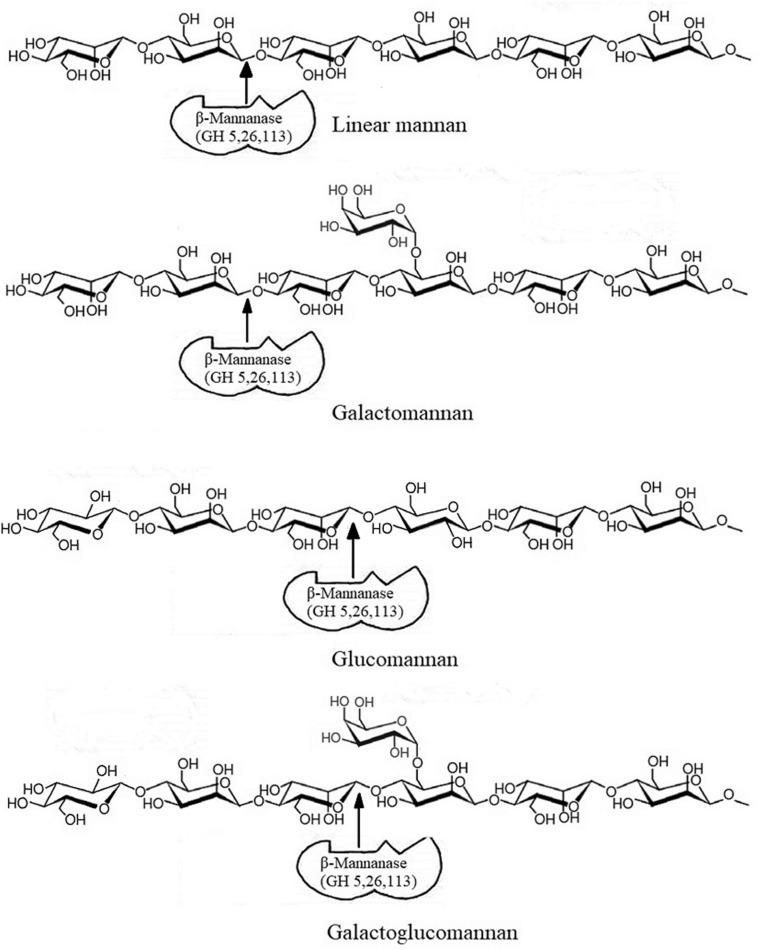
Action of β-mannanase on different types of mannan. Modified from Yeoman et al. (2010).

In grasses and hardwoods, xylan is the major hemicellulose component, while in softwoods, plant fruits and seeds the hemicellulose is mainly present in the form of mannan (Scheller and Ulvskov, 2010). Mannan mainly appears in four different forms: linear mannan, galactomannan, glucomannan, and galactoglucomannan. β-mannanase is a major mannan degrading enzyme (Figure 1). However, due to heterogenous nature of mannan, its biodegradation may require a close association and synergy among β-mannanase (EC 3.2.1.78), β-mannosidase (EC 3.2.1.25), acetyl mannan esterase (EC 3.1.1.6), β-glucosidase (EC 3.2.1.21), and α-galactosidase (EC 3.2.1.22) to break the main and the side chains of mannan (Chauhan et al., 2012; Malgas et al., 2015).

Mannan-degrading enzymes are classified into different glycosyl hydrolase families (like GH 1, GH 2, GH 3, GH 5, GH 26, GH 27, GH 113, etc.) catalyzing the production of oligosaccharides and monosaccharides that can be used for microbial metabolism. These enzymes can also enhance plant metabolism, such as ripening and maturation by metabolizing mannan present in cell wall (Morreira, 2008).

**FIGURE 2:**
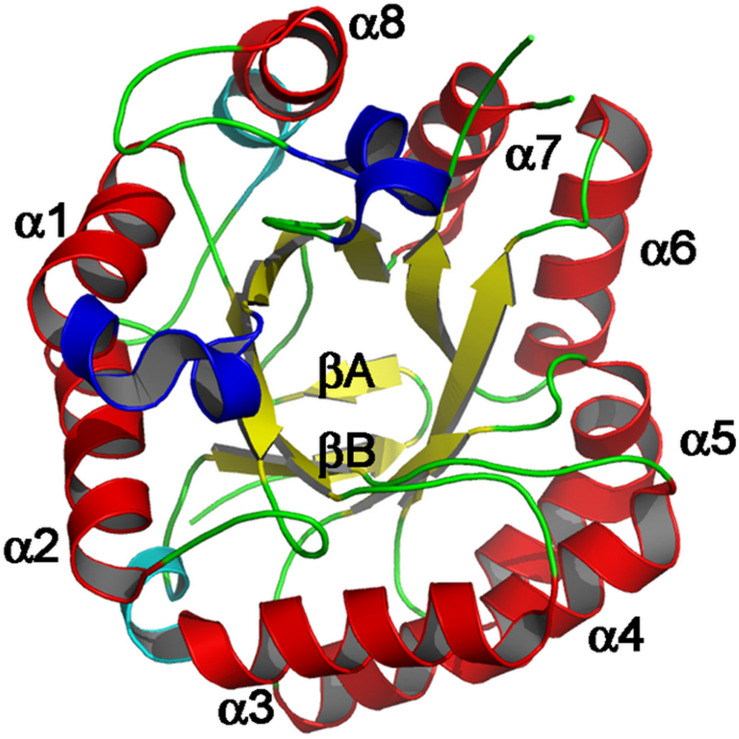
Schematic representation of modular structure of β-mannanase, displaying the Tim barrel structure. Major α-helices are colored red while β-strands are shown as yellow, labeled in accordance with the ideal (β/α)8 barrel structure. Adopted with permission from Zhao et al. (2011).

The primary structure of mannanases in different GH families is different but they are similar in their spatial arrangement, (β/α)8-barrel protein folds and are assembled into clan GH-A (Srivastava and Kapoor, 2017). Mannanases often exhibit modular structure consisting of carbohydrate binding module(s) (CBM), catalytic domain(s), and additional functional domain(s) (Figure 2; Sunna, 2010).

**FIGURE 3:**
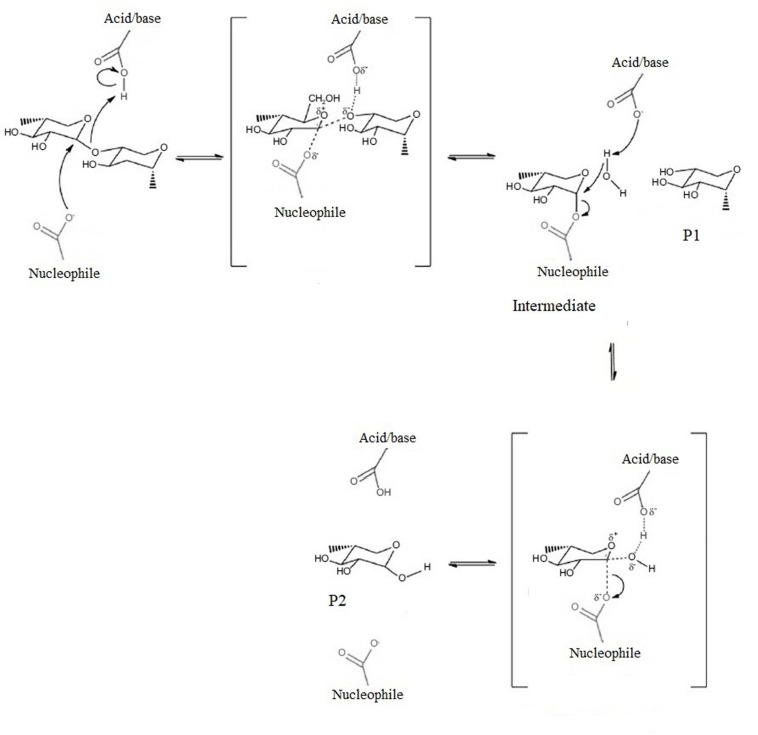
Reaction mechanism of β-mannanase. Modified from Sharma et al. (2018). The brackets indicate transition state. P1 is product 1, P2 is product 2.

Site-directed mutagenesis and X-ray crystallographic studies in a broad variety of species have showed that β-mannanase needs a minimum of five substrate binding sites and a cleft shaped active site with nucleophile catalyst and a well conserved acid/base catalyst which are at a distance of 5.5 Å from each other for efficient hydrolysis of substrates (Figure 3; Hogg et al., 2003; Yan et al., 2008; Tailford et al., 2009; Songsiriritthigul et al., 2011).

Recently β-mannanases have attracted significant attention from both industry as well as academia because of their potential applications in many important sectors of industry including oil drilling, detergents, textile, food, animal feed, and production of bioethanol. This paper presents an in-depth review of potential applications of β-mannanase in light of recently reported studies (2015–2020). Engineering of β-mannanases to efficiently meet industrial needs is also discussed along with a brief but comprehensive discussion of β-mannanase occurrence, purification, and yield improvement.

However, the present review does not discuss in detail the structure, reaction mechanism, assay methods, and biochemical characteristics of β-mannanases. For this the readers are directed to earlier reviews (Dhawan and Kaur, 2007; Chauhan et al., 2012; Srivastava and Kapoor, 2017).

## Microbial β-Mannanases

Mannanases have been isolated from plants, animals and microorganisms (Kim et al., 2013; Wang et al., 2015; Jana et al., 2018). Most of the commercial β-mannanases have been produced from microbes due to their higher stability, production within limited time and space, cost effectiveness and ease of genetic manipulation (Table 1). This increases their market value and makes them suitable candidates for applications in industry.

**TABLE 1:** Commercial production information of β-mannanases.

Company	Country	Mannanase source	Trademark
ChemGen	United States	*Paenibacillus lentus*	Hemicell^®^
CTC Bio Inc	Seoul, South Korea	*Bacillus lentus*	CTCzyme^®^
Sunson	China	*Bacillus lentus*	Nutrizyme^®^
Novo Nordisk	Denmark	*Aspergillus niger*	Gamanase^®^
Advanced Enzymes	India	N. A	DigeGrain M
Novozymes	Denmark	*Talaromyces leycettanus*	Mannaway
Aumgene Biosciences	India	N. A	Mannazyme XP^®^
PhylloZyme	United States	*Trichoderma reesei*	Cp-mannanase
Genencor International	United States	N.A	MannaStar^TM^
Megazyme	Ireland	*Aspergillus niger*	E-BMANN
Genencor International	United States	Fungal β-mannanase	Purabrite
Diversa Corp.	United States	*Thermotoga* sp.	Pyrolase160
Diversa Corp.	United States	*Thermotoga* sp.	Pyrolase200

In the microbial world, numerous microbes possess the ability to degrade mannan efficiently (Table 2). Among bacteria, most of the mannan degraders are gram positive bacteria such as *Bacillus* species (David et al., 2018). But there also some gram-negative bacteria like *Klebsiella Oxytoca* that also efficiently degrade mannan (Tuntrakool and Keawsompong, 2018). Competent mannan degraders among fungi are the members of genus *Aspergillus* while β-mannanases have also been isolated from *Trichoderma* sp. and *Penicillium* sp.(Agrawal et al., 2011; Blibech et al., 2011; Liu et al., 2020). Among actinomycetes *Streptomyces* sp. and *Nocardiopsis* sp. have shown appreciable mannan degrading ability (Gohel and Singh, 2015; Pradeep et al., 2016).

**TABLE 2:** Sources of β-mannanases*.

Sources	References
**Bacteria**	
*Bacillus sp. SWU60*	Seesom et al., 2017
*Bacillus subtilis subsp. inaquosorum CSB31*	Regmi et al., 2017
*Gloeophyllum trabeum CBS900.73*	Wang et al., 2016
*Lactobacillus casei HDS-01*	Zhao et al., 2020
*Klebsiella pneumonia SS11*	Singh et al., 2019
*Bacillus pumilus GBSW19*	Zang et al., 2015
*Bacillus nealsonii PN-11*	David et al., 2018
*Bacillus subtilis YH12*	Liu et al., 2015
*Bacillus sp. MK-2*	Zhang et al., 2019
*Bacillus* sp. R2AL2A	Kim et al., 2018
*Klebsiella oxytoca KUB-CW2-3*	Tuntrakool and Keawsompong, 2018
*Bacillus subtilis P2-5*	Pangsri and Pangsri, 2017
*Bacillus clausii strain S10*	Zhou et al., 2018
**Fungi**	
*Talaromyces trachyspermus B168*	Ichinose et al., 2017
*Lichtheimia ramosa*	Xie et al., 2019
*Aspergillus kawachii IFO 4308*	Liu et al., 2020
*Trichoderma longibrachiatum RS1*	Ismail et al., 2019
*Rhizopus microsporus*	Li et al., 2020
*Aspergillus nidulans*	von Freiesleben et al., 2016
*Aspergillus terreus FBCC 1369*	Soni et al., 2016
*Aspergillus niger CBS 513.88*	Tang et al., 2019
*Aspergillus oryzae RIB40*	Tang et al., 2016
**Other**	
*Streptomyces Sp. CS147*	Yoo et al., 2015
*Streptomyces Sp. CS428*	Pradeep et al., 2016
*Nocardiopsis xinjiangensis strain OM-6*	Gohel and Singh, 2015

*Only representative references published after 2015 are included. For more information, the readers are advised to refer to Dhawan and Kaur (2007), Chauhan et al. (2012), Srivastava and Kapoor (2017).

## Production, Yield Improvement and Purification of β-Mannanases

β-mannanases are mostly produced by using submerged fermentation. However, in a few studies β-mannanases have been produced in solid state fermentation. A number of nutritional and physico-chemical factors like temperature, incubation time, pH, carbon source, and nitrogen content affect the production of β-mannanase. These factors are different for different microorganisms. For example, the incubation time for β-mannanase production in *Acinetobacter* sp. ST 1 is 24 h (Titapoka et al., 2008) while in *Bacillus nealsonii* PN-11 is 96 h (Chauhan et al., 2014). In contrast, the incubation time in fungi usually ranges from 6 days in case of *Penicillium occitanis* (Blibech et al., 2011) to 11 days by *Aspergillus* ATCC 20114 (Mohamad et al., 2011). In majority of the cases the optimum temperature for β-mannanase production lies in mesophilic range, which is closely related to the growth temperature required for that microorganism. In general, for growth and β-mannanase production, fungi usually prefer acidic pH while bacteria prefer neutral to alkaline conditions (Chantorn et al., 2013; Yopi et al., 2017). Several studies have shown enhanced production of β-mannanase through process optimization. Approaches such as one factor at a time have been employed to improve production (Olaniyi et al., 2013; Khattab et al., 2020). Statistical methods like central composite design, Box Behnken and placket Burman or their combination have also been used to enhance the production of β-mannanase (Abd Rashid et al., 2012; Yatmaz et al., 2016; Jana et al., 2018; Blibech et al., 2020).

In addition, in a number of cases, β-mannanase production have also been increased through heterologous expression systems to meet certain industrial needs and ensure economic feasibility of the process.

An engineered acidophilic and thermophilic β-mannanase (ManAK) derived from *Aspergillus kawachii* strain IFO4308 is overexpressed in *Pichia pastoris* (Liu et al., 2020). By means of high cell density fermentation, a maximum yield of 11,600 U/mL and 15.5 g/L are obtained, which is greater than majority of β-mannanases. However, the highest titers of β-mannanase have been reported from *Rhizomucor miehei* (79,680 U mL^–1)^ and *Chaetomium* sp. CQ31 (50,030 U/ml) following cloning and expression of their β-mannanase enzyme gene in *Pichia pastoris* (Katrolia et al., 2012; Li et al., 2018).

Heterologous expression of β-mannanase in *Pichia Pastoris* has been used widely, however, *Saccharomyces cerevisiae* expression system has also been reported to be safe and effective. A β-mannanase gene derived from *Aspergillus sulphureus* is optimized and expressed in five different *S. cerevisiae* strains and the properties of the strains are evaluated (Liu et al., 2018). Haploid strain BY4741 integrated with β-mannanase gene under constitutive promoter TEF1 shows the highest efficiency expression. After 36 h the enzyme activity reaches ∼ 24 U/ml and production efficiency 16 U/mL/day.

Depending on properties of β-mannanases, different protocols related to purification of mannanase often involve the use of 2–4 chromatographic steps (Table 3).

**TABLE 3:** Protocols used to purify β-mannanases from various microorganisms.

Organism	Purification protocol	No. of steps	Yield (%)	Purification fold	Specific activity (U/mg)	References
*Weissella viridescens* LB37	(NH4)2SO4, DEAE-Sephadex, Sephacryl S-200	3	20.5	188.07	63.94	Adiguzel et al., 2016
*Bacillus* sp. CFR1601	(NH4)2SO4 (50–80%), DEAE-Cellulose, DEAE-Sepharose and Phenyl-Sepharose	4	21.3	50.7	10,461.5	Srivastava and Kapoor, 2016
*Bacillus nealsonii* PN11	(NH4)2SO4 (60–80%), Sephadex G-150, and DEAE-Cellulose	3	8.92	38.96	2280.9	Chauhan et al., 2014
*Klebsiella pneumoniae* SS11	(NH4)2SO4 (50–80%) Sephacryl S-200	2	9.6%	5.50	7573.57 IU/mg	Singh et al., 2019
*Bacillus* sp.CSB39	Sepharose CL-6B, DEAE Sepharose Fast Flow	2	25.47	19.32	1063.91	Regmi et al., 2016
*Enterobacter ludwigii*	(NH4)2SO4 (30–60%), Sephadex G-25, DEAE cellulose, Sephadex G-100	4	15.16	11.71	4510.37	Yang et al., 2017
*Lactobacillus plantarum* (M24)	(NH4)2SO4 (60–80%), DEAE-Sephadex, Sephacryl S-200	3	14.7	2619.05	82.5	Adiguzel et al., 2015
*Pholiota adiposa* SKU0714	MWCO 10 kDa, Gel filtration chromatography	2	9.99	57.2	1,870	Ramachandran et al., 2014

## Industrial Applications of β-Mannanases

There is a wide range of industrial applications of β-mannanases in various areas. The following will describe some examples including use in food, pulp, energy production, textile, detergent, animal feed, and pharmaceuticals.

### Use in Oil Drilling

Due to an ever-widening gap between global demand and supply of energy, well fracturing is frequently carried out for the recovery of oil and gas. Following fracturing of well formations, controlled breakage of fluid is required to facilitate enhanced recovery of the trapped gas or oil.

In drilling operations β-mannanase application can be useful. To maximize product flow, now a days, guar gum along with sand particles is used to flood the well followed by pressurizing of the bedrock until it breaks open (Thombare et al., 2016). In order to ease the flow of the product from the well, the polymer solution must be thinned out and β-mannanases that can bring about hydrolysis of guar gum at high temperature (>80°C) can be useful for this purpose.

β-mannanases with high temperature optima are particularly suited for oil drilling operations because of the presence of geothermal gradients in deep oil wells. The use of such enzymes prevents the hydrolysis of guar gum at the earth’s surface while only the environmental temperature at the depth of the well are suitable for the enzyme activity. Considerable research efforts have been directed toward the isolation and characterization of such β-mannanases. A novel β-mannanase (DtManB) from *Dictyoglomus thermophilum* has been reported to be a suitable candidate for oil drilling operations because it shows excellent activity and stability at high temperature (80°C) and low activity at low temperature (Hu et al., 2014). DtManB effectively reduces the viscosity of hydroxypropyl guar solution, even in the presence of different additives. In addition, DtManB could adequately break cross linked fracturing fluid. However, the increase in optimum temperature of DtManB to even higher degree (>90°C) through enzyme engineering could make it an even more desirable candidate.

Similarly, another β-mannanase suitable for oil drilling operations has been discovered in a hydrothermal vent sample that can prevent rehealing of gel by effectively breaking linear and borate cross linked guar polymers into small soluble fragments (Zhang et al., 2013). The enzyme shows excellent activity between 60 and 80°C. During fracturing operations, the enzymatic reaction can be activated by a change in temperature and pH of the environment. A good dose response has also been displayed by this β-mannanase which may enable the user to adjust the dosage of the enzyme to achieve a desired viscosity/time profile. In addition, it has been reported that this superior β-mannanase effectively reduces viscosity even in the presence of different additives such as salts, buffers, cross linkers and stabilizers. Thus, making this β-mannanase very suitable for effective viscosity reduction in oil drilling operations.

The selection of the enzyme for breaking the guar gel should be made on the basis of well temperature so as to maximize the performance of the enzyme. Some oil wells have low temperature and for those low temperature active β-mannanase can be effective.

A halotolerant, low temperature active β-mannanase isolated from a novel strain of *Enterobacter* sp. N18 is purified and tested for its potential in hydrolyzing fracturing fluid based on guar gum (You et al., 2016). Within 10 min, the viscosity of the fracturing agent was reduced by more than 95%. The enzyme has a temperature optimum of 50°C and at 20°C it retains 50% of its activity, showing that β-mannanase from strain N18 could be very effective in oil wells where the temperature is low and chemical gel breaking agents are inactive.

As the temperature of most oil wells can be higher than 80°C, many fungal mannan-degrading enzymes do not find use in oil drilling operations. However, TtMan5A, a thermostable β-mannanase derived from fungus *Talaromyces trachyspermus* has an optimal temperature of 85°C and good catalytic activity in a wide range of pH (Ichinose et al., 2017). TtMan5A could be an excellent candidate for use in drilling operations.

A few β-mannanases with high thermostability have appeared on the market showing potential for enhanced viscosity reduction, leading to increased production as well as revenue per well. Mannanases marketed by Diversa (San Diego, CA, United States) under the trade name Pyrolase are the typical examples. Isolated from the nature and screened for their high thermostability, Pyrolase160 and Pyrolase 200 have shown excellent performance in drilling operations.

### Fruit Juice Clarification

In recent years, the preference for natural fruit juice consumption has increased in health-conscious consumers. Raw fruit juice, however, is viscous and turbid and typically settles during storage (Nagar et al., 2012). To market it, it must be clarified first. The viscosity and turbidity of natural fruit juice is mainly due to pectin, starch, cellulosic and hemicellulosic compounds (Dey and Banerjee, 2014; Sharma et al., 2014). For processing of these polysaccharides, microbial enzymes are used which hydrolyze these compounds and this results in a juice that is more suitable for the consumers taste, in addition to having good storage stability. The amount and composition of these polysaccharides varies in different fruits so different enzymes are needed for their processing. Compared to the use of chemicals the enzymatic process is advantageous because it has higher specificity, requires mild reaction conditions and produces less waste (Sharma et al., 2014). The addition of β-mannanase has shown improvement in fruit juice clarification and increased yield. Different studies have investigated the effect of β-mannanase to reduce the viscosity and turbidity of different juices like apples, grape, kiwi, peach, pomegranate, and orange.

β-mannanase hydrolyzes the mannan fraction of the hemicellulose present in fruit juice thus reducing its viscosity and releasing the water trapped within it. Thus, β-mannanase hydrolysis also increases the amount of fruit juice. A β-mannanase isolated from *Weissella viridescens* LB37 was effective in juice clarification from different fruits, with a ratio of 112.7%, 108.97%, 107.8%, 110.0%, 117.21%, 109.2% in grape, peach, orange, pomegranate, kiwi, and apple, respectively, as compared to control (Adiguzel et al., 2016). In another study, a thermo-alkaline β-mannanase from *Bacillus pumilus* (M27) was isolated from a sausage sample (Adiguzel et al., 2015). This β-mannanase is highly stable at a range of temperature (30–80°C) and pH (3–11). Among different fruit juices tested, the highest yield at the rate of 154% was obtained when β-mannanase enzyme was used to clarify apple juice.

Filtration and centrifugation can also be employed in fruit juice clarification, but enzymatic process is superior because it has high efficiency. In a study reported by Zhao et al. (2019)β-mannanase is isolated from *Lactobacillus casei* HDS-01 with high biosafety level and its potential in fruit juice clarification is tested. β-mannanase treatment of fruit juice results in a significant increase in clarity and yield of different fruit juices. Pear juice clarified from 54.32 to 65.28%, orange juice from 39.50 to 46.30%, and apple juice from 57.20 to 69.25%. In terms of juice yields represented by volumes, *L. casei* HDS-01 crude β-mannanase treatment produces apple, orange and pear juice with a yield ratio of 132.5%, 156.00%, and 151.04%, respectively compared to control.

Further purification of crude β-mannanase in this study is not considered necessary because of the high biosafety level of *Lactobacillus casei* HDS-01 strain. Thus, β-mannanase is not separated from bacterial cells to clarify juices. The use of crude β-mannanase can be more advantageous because enzyme purification requires time and cost. However, when purified enzyme is used the yield and clarity of juice increases further (Zhao et al., 2020). For orange juice clarification, the application of the purified *L. casei* HDS-01 β-mannanase when compared with control results in a clarity of 47.55 ± 0.02% and yield of 188.20 ± 0.40%. For apple juice clarification, treatment with HDS-01 β-mannanase achieves the clarity of 72.30 ± 0.04% and yield of 150.96 ± 0.40%.

### Bio-Bleaching of Pulp

Bleaching is the treatment of cellulosic fiber with chemicals to increase brightness which can be achieved through lignin removal or lignin decolorization (bleaching). This may require the use of a lot of chemicals which are hazardous to environment. However, the process can be made more environmentally friendly by using hemicellulases that open up the fiber structure, preparing the lignin to be removed away rather easily and thus reduce the amount of chemicals used in the subsequent steps.

Majority of pulp is derived from softwood which contains about 15–20% hemicellulose present as galactomannan (Chauhan et al., 2012). β-mannanase enzymes that are highly specific for galactomannan substrate thus can make good candidates for use in pulp and paper industry.

Patel and Dudhagara (2020) report that rice straw pulp can be effectively bleached by first treating it with an enzyme cocktail (xylanase, β-mannanase, pectinase) and then with diluted chemicals (DC) instead of a sole application of either the enzymatic mixture or DC. Treatment of rice straw pulp with both DC (0.25% EDTA and 0.5% H_2_O_2_) and enzyme cocktail leads to an increase in the release of phenolic compounds, reducing sugars and hydrophobic compounds and reduction in Kappa number. Based on these observations, it is concluded that pretreatment of pulp with an enzymatic mixture derived from *Isoptericola variabilis* UD-6 can decrease the quantity of chemicals employed in the process, thus making the process cost effective and environmentally friendly.

Enzymes that have high thermostability and are active at a broad range of pH are of special interest in pulp and paper industry because of the high pH and temperature conditions applied. This is why research efforts have been directed toward discovering β-mannanases that are remarkably alkali tolerant and thermostable. In Kraft pulping an additional important feature is required which is that the enzyme should be resistant to both alkaline and neutral proteases. A good example is Man5A that has been reported to retain its catalytic efficiency more than 97% following treatment with chymotrypsin, trypsin, collagenase, proteinase K, and subtilisin A for 30 or 60 min (Luo et al., 2012). Similarly, in another study, a multi-tolerant thermostable β-mannanase (MnCSB39) from *Bacillus sp.* CSB3 is isolated and characterized (Regmi et al., 2016). In addition to being active at a wide pH and temperature range the enzyme is also urea, NaCl, surfactant, and protease tolerant. The increased halotolerance of MnCSB39 makes it very suitable for bio-bleaching of pulp, where the concentration of Na^+^ and Cl^–^ can be very high. Worth mentioning are a few other multi-stress tolerant enzymes such as Man5p1 isolated from *Neosartorya fischeri P1* and rMan5HJ14 isolated from *Bacillus* sp. HJ14 and MnB31 from *Bacillus inaquosorum* CSB31 which are resistant to urea, NaCl, Ag^+^ ions, SDS as well as the action of proteases (Yang et al., 2015; Zhang et al., 2016; Regmi et al., 2017).

The combination of β-mannanase with other hemicellulases may augment the positive effects of β-mannanase application. Yang et al. (2017) observes that significant improvement occurs in brightness of kraft pulp when β-mannanase is coupled with xylanase.

### Hydrolysis of Coffee Extract

**FIGURE 4:**
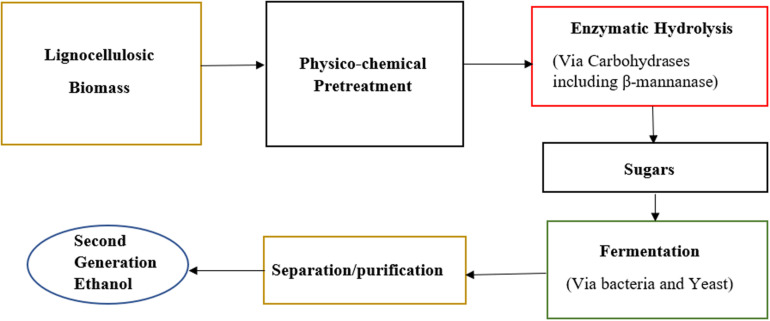
Overview of bioethanol production from lignocellulosic biomass based on β-mannanase hydrolysis.

Instant coffee provides its consumers with combined advantages of convenience and high added value. The main residue produced during instant coffee production is spent coffee ground (SCG) which consists mainly of polysaccharides like galactomannan and cellulose (Jooste et al., 2013). These polysaccharides do not get solubilized during the extraction process and therefore are left as insoluble solids (Figure 4).

In this backdrop, application of β-mannanase is seen as a favorable strategy for solubilizing/hydrolyzing remaining solids, thereby increasing yields of soluble solids of instant coffee. Jooste et al. (2013) applied different carbohydrase enzymes to enhance solubilization of remaining solids produced during coffee production. Among the enzymes tested for the hydrolysis of SCG, the highest increase in soluble solids yield is obtained by use of β-mannanase (Man 1). Combining β-mannanase (Man1) with other enzymes shows an additive effect instead of a synergistic one which indicates that β-mannanase is mainly responsible for the highest soluble solids yield. Similarly, Favaro et al. (2020) also demonstrate that a large amount of carbohydrates in the SCG can be hydrolyzed by β-mannanase. The hydrolysis yield increases even more (56%) when a commercial cellulase cocktail is added to β-mannanase, demonstrating the promise to increase soluble coffee processing. In a study reported by Baraldi et al. (2016) enzymatic and thermal processes are compared during the production of instant coffee. Roasted coffee is first extracted with water at 125°C and the spent coffee is then processed by either enzymatic hydrolysis at 50°C (with the aid of a cocktail of enzymes containing β-mannanase) or thermal hydrolysis at 180°C. Enzymatic hydrolysis yield (18%) is lower than thermal hydrolysis yield (28%). But instant coffee produced through enzymatic hydrolysis not only has low amount of unwanted compounds like furfural, acetaldehyde and 5-HMF but also less energy is consumed in the process. These findings demonstrate that the enzymatic procedure is a viable substitute to thermal hydrolysis for the production of instant coffee.

### Bioethanol Production

**FIGURE 5:**
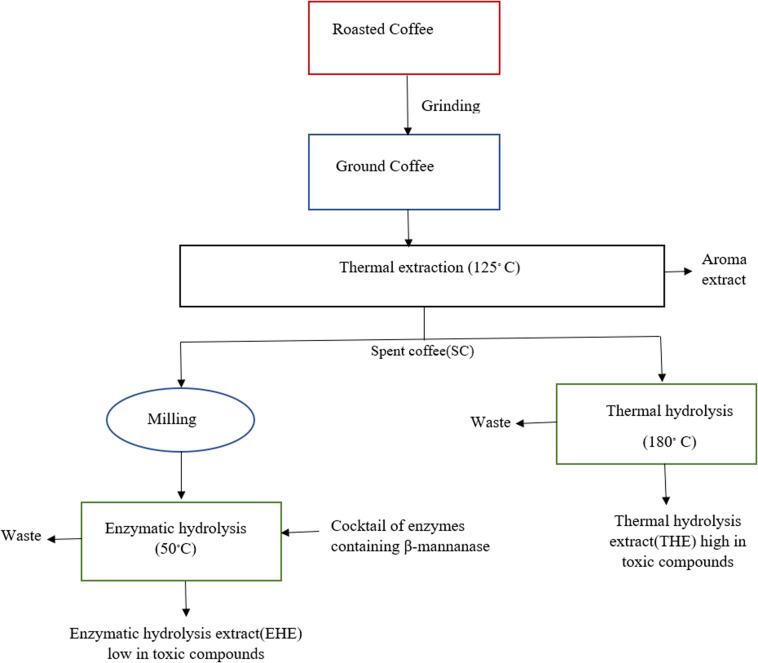
Comparison of soluble coffee production by enzymatic and thermal hydrolysis. Modified from Baraldi et al. (2016).

Ethanol production can be divided into first generation and second-generation processing. Fermenting food-biomass into ethanol in a process similar to wine and beer making produces first generation ethanol. Second generation ethanol is produced by fermentation of lignocellulosic materials by microorganisms, however, this requires pretreatment because of the recalcitrance of plant cell wall (Zhang, 2019). Once the recalcitrance of plant cell wall is reduced through pretreatment, the hydrolytic enzymes of the microorganisms can act on cell wall polysaccharides and hydrolyze them to sugars monomers. As mannan is a component of lignocellulosic materials, supplementation of mannan-degrading enzymes can be beneficial in the production of mannose sugar which is then fermented by yeast into second generation ethanol (Figure 5).

A promising mannan rich bioresource is palm kernel cake (PKC) that can be used for fermentable sugar extraction. Hydrolysis of PKC by β-mannanase is increased under optimum conditions where β-mannanase loading, time, pH, temperature, and PKC concentration are optimized (Shukor et al., 2016). A total of 71.54 ± 2.54 g/L of fermentable sugars are produced under optimized conditions. This sugar hydrolysate is fermented by *Clostridium saccharoperbutylacetonicum* N1-4, producing a total of 3.27 ± 1.003 g/L of biobutanol.

In another study a bio-engineered mannanase (mRmMan5A) derived from *R. miehei* is successfully used for mannooligosaccharides production from steam explosion pretreated PKC (Li et al., 2018). More than 80% of PKC mannan is converted into mannooligosaccharides by the hydrolytic action of mRmMan5A. In addition, to ascertain the feasibility of mass production, kilogram scale of mannooligosaccharide production is also carried out and a yield of 26.1% is obtained, proving mRmMan5A is an efficient β-mannanase for the bioconversion of mannan rich biomass.

β-mannanase is an important enzyme for hydrolysis of mannan. When it degrades mannan it mainly produces oligomers along with a small amount of mannose. These oligomers or mannooligosaccharides (MOS) need to be degraded further by mannosidases to produces monomers which can then be utilized by ethanologens. In a study reported by Raita et al. (2016) a thermophilic bio-engineered ethanologen *Geobacillus thermoglucosidasius* TM242 is used to produce ethanol directly from MOS rich sugar hydrolysate of PKC. Without needing mannosidase, *G. thermoglucosidasius* TM242 produces a maximum concentration of ethanol of 9.9 ± 0.4 g/L, which is equivalent to 92 ± 2% of theoretical yield based on the total convertible sugars present in PKC. This yield of ethanol is much greater than when *S. cerevisiae* is used under the same conditions to hydrolyze PKC. For the production of ethanol from mannan rich waste, use of ethanologens like *G. thermoglucosidasius* TM242 seems highly attractive as mild pre-treatment conditions are required and all sugars and oligosaccharides present in the substrate can be utilized without the need of β-mannosidase supplementation in hydrolysis step.

Effectiveness of β-mannanase for bioconversion of lignocellulosic biomass increases when used in combination with other carbohydrase enzymes. Addition of β-mannanase (MtMan26A) from thermophilic fungus *Myceliophthora thermophila*, to commercial enzyme mixture (Novozymes^®^ 188 and Celluclast^®^ 1.5 L) effectively hydrolyzes pretreated beechwood sawdust, increasing the release of total sugars by 13% and glucose by 12% (Katsimpouras et al., 2016). Similarly Cerveró et al. (2010) report that the conversion of lignocellulosic biomass into monosaccharides could be increased by using a binary mixture of commercial enzymes in the ratio of 1:1. Combining commercial enzymes, i.e., Mannaway (mannanase) and Gammanase (mannanase and galactosidase) shows an excellent synergistic effect that releases 30% more mannose than when those enzymes are used individually. Zhang and Sang (2015) also demonstrate that it is more effective to use a cocktail of enzymes to produce fermentable sugars from lignocellulosic biomass.

### Textile Industry

β-mannanases in combination with detergents can be used for cleaning or preparation of fibers in textile and cellulosic processing industries. To prepare the material that is ready for garment manufacture the cellulosic material is processed through several steps: singing, desizing, scouring, bleaching, dyeing, and finishing (Mojsov, 2011). Application of β-mannanase can be useful in the bio-scouring and desizing of cellulosic fibers, thus preparing the material for good response in subsequent dyeing operations.

Bio-scouring is the process in which the fabric is cleaned through enzymatic action from impurities like pectin, hemicelluloses, wax, and mineral salts (Bristi et al., 2019). These impurities make the raw cotton hydrophobic and thus interfere with aqueous chemical process like dyeing and finishing. Therefore, removal of these impurities is essential before the fabric can be dyed.

### Detergent Additives

Application of microbial enzymes in the detergent industry is well known. Among them, the most frequently used enzymes are proteinases, lipases, amylases, and cellulases (Srivastava and Kapoor, 2014). Lately, alkaline β-mannanases which show stability toward detergent components are increasingly being used as stain removal boosters in certain laundry segments. β-mannanase hydrolyzes different mannan based materials such as guar gum, glucomannan, galactomannans and others (Liao et al., 2014). Present as thickeners and for their gel textures, guar gums are found in an increasing number of consumer products, including barbecue sauce, ice cream, salad dressing, makeup, and hairstyling (Mudgil et al., 2014). These gums act like glue and stick to soil particles, thus making it difficult to remove dirt. β-mannanases effectively hydrolyze these gums, removing it from the fabric and thus preventing the dirt from sticking to the fabric.

ManSS11, a β-mannanase enzyme isolated from a novel *Klebsiella pneumoniae* strain SS11 was used to conduct wash performance experiments (Singh et al., 2019). Analysis of the hydrolyzed products, done at the end of washing process, shows that β-mannanase had 80.5 ± 1.07% better detergency (cleansing power) in removing locust bean gum + dust fixed strain than when detergent is used alone which shows only 30.6 ± 0.86% detergency. Similarly, in another study, cleaning power of β-mannanase derived from *Bacillus* sp. CFR1601 is tested against a cotton cloth stained with chocolate-ice cream and tomato sauce (Srivastava and Kapoor, 2014). Detergent combined with β-mannanase shows enhanced stain removal than sole application of detergent. In addition, the stability and compatibility of the isolated β-mannanase with different laundry detergents available in the market, has also been checked. The enzyme reserves 89.0–100% of its original activity at 37°C for up to 1 h in the presence of different detergents in the sequence: Wheel > Surf > excel > Ariel > Tide > Rin. This shows that performance of the enzyme is influenced by the ingredients present in the detergent, as enzyme stability varied with different detergents tested. In a study reported by David et al. (2018) optimization of co-production of protease and β-mannanase from a *Bacillus nealsonii* strain PN-11 is investigated along with the potential of both of these enzymes as additives to detergents. The enzymes show good compatibility with detergents and the detergent performance is improved on different kinds of stains, when either β-mannanase or protease is used. However, the destaining is much more efficient when both β-mannanase and protease are used in combination.

For application in detergent industry, the enzyme should be highly thermostable and active in broad range of pH like the case of β-mannanase isolated from *Bacillus halodurans* PPKS-2. This enzyme is reported to be extremely alkaliophilic, halotolerant, detergent, and thermostable (Vijayalaxmi et al., 2013). It has an optimum temperature of 70°C and retains 100% of its original activity at 70°C for up to 3 h. Its optimum pH is 11 and the enzyme is active in a wide range of NaCl concentrations (0–16%). These properties make it a suitable candidate for the detergent industry. Another extremely alkaline, thermostable β-mannanase is isolated from *Streptomyces* sp. CS428 which effectively hydrolyzes galactomannan and thus can be effective in removing guar gum stains (Pradeep et al., 2016). Similarly, RmMan5A is especially suitable for use in detergent industry, due to its excellent stability in a wide range of pH (4.0–10.0) and thermostability up to 55°C (Katrolia et al., 2013). In addition, it displays remarkable tolerance toward sodium dodecyl sulfate (SDS), which has been reported to impede the activity of several other β-mannanases.

There are opportunities for geographical and numerical extension of β-mannanase use in detergents. In developing countries β-mannanases have not found a wide spread use, even though these countries are dusty and hot and thus frequent washing of clothes is often needed. In west, particularly in United States, β-mannanase has found its way in commercial household detergents preparations. Novozymes, a Danish company, markets Mannaway which is a washing detergent containing β-mannanase. This can effectively be used for removal of mannan based stains. The inclusion of β-mannanase in detergents not only improves the stain removal ability of detergent but also prevents other particles from sticking to the fabric during washing process.

Purabrite is another commercial β-mannanase marketed by another United States company Genencor. For decades, detergent formulators have faced the main challenge of developing products with superior cleaning performance at competitive prices. Genencor claims that Purabrite meets these requirements. Purabrite is available in both liquid and granular form. The granular shape including a patented Enzoguard^®^ coating is a proprietary technology of Genencor. These granules are safer to handle than poly powders and have enhanced properties for easy mixing and storage.

### In Animal Feed Industry

β-mannanase supplementation in diet especially in high fiber diets or low energy diet has been reported to benefits the animals in many different ways.

β-Mannanase enhances growth performance and ileal digestible energy (IDE) and decreases intestinal viscosity in broilers fed diets with varying levels of galactomannan (Latham et al., 2018). The observed beneficial effects of β-mannanase are considered to be contingent upon concentration of dietary galactomannan. In a study carried out on turkeys β-mannanase supplementation had a beneficial effect on jejunum mucosal morphology (Ayoola et al., 2015). The villus tip width increased by 36%, villus height/crypt depth by 32%, villus surface area by 34%, and base width by 22.5%. Besides improving gut morphology Ayoola et al. (2015) observed that β-mannanase supplementation also causes thinning of ileal mucin layer. Increased mucin secretions have been linked to proliferation of intestinal pathogens. Thus, a reduction in mucin secretions may reduce the risk of pathogen proliferation in the gut and help establish symbiotic enteric ecosystem. β-mannanase supplemented with a cocktail of enzymes has also shown to be beneficial. Govil et al. (2017) observes that β-mannanase supplementation in combination with other carbohydrase enzymes shows significant improvement in feed conversion efficiency, weight gain and performance index in broilers.

Copra meal is easily available in many parts of the world and can be a cheap alternative to commonly used feed ingredients like SBM and corn. But its reduced amino acid (AA) digestibility and low energy content can be a barrier to its frequent use. Kim et al. (2017) demonstrate that copra meal if supplemented with β-mannanase (800 IU) can replace corn and SBM up to 25% without negatively affecting the growth performance and pork quality in growing finishing pigs. El-Masry et al. (2017) observes that addition of 5% guar meal (GM) in broiler diet has deleterious effect on growth performance. But with supplemental β-mannanase GM can be used at 5% without adversely affecting blood biochemistry and growth.

Very few studies have been conducted on β-mannanase supplementation in aquaculture. Chen et al. (2016) observes that β-mannanase supplementation (0.5 and 1.0 g/kg) in a plant based diet to tilapia significantly improved weight gain, FCR and specific growth rate indicating that β-mannanase is beneficial for tilapia.

### Production of Mannooligosaccharides (MOS) as Immune Modulators, Anti-Tumor and Weight Loss Agents

Mannan is degraded into MOS by β-Mannanase. MOS are a kind of prebiotics and have been reported to boost the growth of beneficial gut microbiota like *Lactobacilli* and *Bifidobacteria*.

These bacteria or probiotics have many beneficial functions. Actually, most of the positive effects of MOS are due to propagation of these beneficial bacteria. Various researchers have studied MOS production by hydrolytic action of β-mannanases from different microorganisms (Pradeep et al., 2016; Rahmani et al., 2017; Yopi et al., 2017; Bååth et al., 2018).

Mannooligosaccharides can function as immune modulators. Atopic allergies have recently increased as dietary habits and environment have changed. Allergic reactions are characterized by an increase in serum IgE antibody, cytokine production (IL-8 and TNF-α) and infiltration of acidophil cells (Galli and Tsai, 2012). Prebiotics can be used to suppress allergic reactions (Brosseau et al., 2019). *In vitro* studies of MOS on ovalbumin-sensitized mice showed a decrease of 56.2 to 36.2 in serum IgE titer. There was also a significant decrease in TNF-α and IL-8 from Peyer’s Patch suggesting that MOS have anti-allergic properties and can be used as anti-allergic agents (Ozaki et al., 2007).

Mannooligosaccharides can be used as anti-tumor agents. Colon cancer in western countries is the second-most common type of cancer (Fernández et al., 2015). The use of prebiotics could lead to more positive effects on the health compared to commercial anti-cancer agents. Prebiotics have been reported to possess anti-cancer properties and detoxify gastrointestinal genotoxins. Recent studies have shown that prebiotics such as MOS have powerful anti-tumor properties. *In vitro* cytotoxicity assay of MOS (500 μg/mL) on human epithelial colorectal adenocarcinoma cell line (HT-29) shows after 48 h, a 60% decrease in the viability of cells (Ghosh et al., 2015). Thus, for colon cancer treatment, MOS can be good therapeutic agents.

Mannooligosaccharides can be used as weight loss agents. Clinical studies have shown that MOS consumption may reduce body weight and thus can be used as an aid to a weight loss regimen. Salinardi et al. (2010) report that in overweight men, consumption of MOS containing drink as part of a weight maintaining diet reduces total body volume and body weight compared to placebo. Similarly, St-Onge et al. (2012) have also observed that adding MOS to a weight loss diet can expedite adipose tissue and weight loss in men suggesting a potential functional usage of MOS in adipose tissue distribution and weight management. However, more research is necessary to further understand the mechanism by which MOS improves the composition of the body and to clarify the influence of gender.

## Bioengineering of β-Mannanases for Improving Their Industrial Applications

To meet industrial needs, an ideal β-mannanase should exhibit certain properties that include high specific activity, good thermostability, activity over a wide range of pH and strong resistance to chemical products and metal cations. As most of the native β-mannanases do not possess optimal properties required by the industry, research efforts have been directed toward bioengineering β-mannanases to enhance their stability and activity.

### Increasing Activity of β-Mannanases

Using crystal structure as basis Huang et al. (2014) apply rational design to an *Aspergillus niger* β-mannanase (ManBK) for increasing its enzyme activity. The most promising mutant (Y216W) produced out of the 23 mutants examined shows an 18 ± 2.7% increase in specific activity compared to the wild type enzyme. The heat tolerance profile and the optimal temperature of both Y216W and the wild type enzyme are similar but the *K*_cat_ values on konjac and guar gum are higher for Y216W, as revealed by the kinetic studies. The enhanced catalytic activity of Y216W is ascribed to the faster dissociation of cleaved sugars which may be due to extended aromatic ring of Trp that changed interaction with bound polysaccharides. By restricting the enzyme from holding the substrate in the binding site the substrate conversion rate is increased.

Suitable substitution of amino acids can facilitate the access of substrate to the substrate binding groove and maintain it therein, thus improving the substrate affinity and catalytic efficiency of the enzyme. In a study by Li et al. (2014) the substrate affinity of AuMan5A is improved by rational modification, which is predicted by *in silico* design, including molecular docking simulations and calculations of binding free energies. Based on this design, the mutant genes *Auman5A*Y111F and *Auman5A*Y115F are created by site-directed mutagenesis. The Auman5A and its mutant genes are expressed in *Pichia pastoris* and characterized. The pH and temperature profile of the expressed mutants is similar to the parental enzyme. However, the *K*_m_ values of reAuMan5AY115F and reAuMan5AY111F for guar gum decrease about 47% and 34%, respectively, and their catalytic efficiencies increase 0.7 and 0.5-fold correspondingly as compared with those of parental enzyme, making them excellent candidates for different industrial processes.

Li et al. (2017b) report that the catalytic efficiencies of a *Rhizomucor miehei* β-mannanase (RmMan5A) under acidic and thermophilic conditions is enhanced. By using error prone PCR and DNA shuffling a mutant library having 0.72% mutation frequency is created. Following two rounds of screening a variant (mRmMan5A) with superior catalytic activity in high temperature and acidic conditions is obtained and characterized. The mutant shows an increase of 10°C in optimum temperature and a 2.5 unit acidic shift in optimum pH compared to the wild type enzyme. Moreover, the catalytic efficiencies (*K_cat_/K_m_*) of mRmMan5A toward different mannan based substrates increase more than threefold in thermophilic and acidic conditions, making it a highly desirable candidate for application in biorefinery industry.

Molecular engineering of Bman26 (a recombinant β-mannanase from *Bacillus* sp. MK-2) is carried out using random mutagenesis in *Bacillus subtilis* WB800, to obtain an enzyme with higher specific activity (Zhang et al., 2019). With the help of error prone PCR, mutant libraries are constructed and three positive mutants with considerably enhanced specific activities are selected and characterized. Among them K291E is the best, in which a single amino acid substitution caused a 3.5-fold increase in *K_cat_/K_m_*. The catalytic efficiency for Konjac glucomannan also increases approximately 80% and 200% for the L211I and Q112R mutants, respectively. Structural and functional analysis of these three positive mutants shows that a small conformational alteration like loss of hydrogen bond between Gln112 and Thr75 in case of mutant Q112R may cause a significant change in substrate binding and certain enzyme characteristics.

A random mutagenesis strategy is used by Couturier et al. (2013) to produce 10,800 mutants of GH5 (PaMan5A) and GH26 (PaMan26A) β-mannanases isolated from the fungus *Podospora anserina.* Further characterization is done of five mutants (PaMan26A-P140L/D416G, PaMan5A-V256L/G276V/Q316H, PaMan5A-36R/I195T/V256A, PaMan5A-K139R/Y223H, and PaMan5A-G311S). Circular dichroism, temperature and pH profiles are like those of their respective parental enzymes. However, compared to the wild type enzymes, all the chosen variants display a significantly enhanced activity. The double mutant PaMan26A-P140L/D416G having mutations at the entry of the active site (D416G) and the linker region (P140L) display an increase of 30% in *K*_cat_/*K*_m_, compared to parental enzyme. The increase in *K_cat_/K_m_* is partially explained by an increase in the flexibility of the linker produced due to P140L mutation, while D416G mutation promotes the entry of the substrate into active site. The Triple mutant PaMan5A-W36R/I195T/V256A displays higher catalytic efficiency with an increase of 1.8-fold and enhanced hydrolysis of galactomannan. With an almost unaltered structure except a slight change in the beta-strand 8, the single mutant PaMan5A-G311S shows an increase in *K_cat_/K_m_* of 8.2-fold, possibly due to a reduction in *K*_m_ because of the positioning of the residue W315 at the surface of the enzyme. These results show that variants with improved catalytic activity can be produced to efficiently hydrolyze softwood biomass to produce MOS and sugar.

To improve the hydrolysis of hemicellulosic biomass, Guo et al. (2013) have created chimeras of β-mannanase and xylanases. The characteristics of β-mannanase and xylanase in chimera and their synergistic abilities to hydrolyze *Luffa cylindrica* fiber efficiently is shown to be affected by the length of linker, the type of linker, and their order of integration.

### Enhancing Thermostability of β-Mannanases

Rational design method is employed to enhance thermostability of β-mannanase (ManTJ102) leading to a mutant with enhanced performance (Wang et al., 2018). With the help of a molecular dynamics simulation, flexible area surrounding residues 330–340 in ManTJ102 is first identified. Then by virtual mutation the critical amino acid residue (Ala336Pro) having the lowest resulting free energy is determined. The mutant produced is named Mutant336. A range of high temperature (50–80°C) is used to experimentally verify the thermostability of both the Mutant336 and the wild type enzyme. Compared to ManTJ102, Mutant 336 shows better thermostability with a half-life at 60°C being 24-fold higher than ManTJ102 and the irreversible thermal denaturation constant is about 2/5 of ManTJ102, making it a highly attractive candidate for MOS production in industry.

Mutations that enhance the thermostability of the enzyme usually lead to reduction in catalytic activity. Therefore, enzyme activity needs to be preserved while improving its thermostability. Zhu et al. (2019) report that directed evolution based on iterative saturation mutagenesis applied to Man25 (a *Thermoanaerobacterium aotearoense* SCUT27 derived β-mannanase) at the five potential Ca^2+^ binding sites lead to improvements in thermostability of the enzyme without decreasing its activity. Out of 7000 clones produced, the best mutant ManM3-3 (D143A) shows enhanced thermostability and satisfactory catalytic activity. Compared to the wild type, its half-life increased 3.6 fold at 55°C.

To enhance the thermostability and catalytic efficiency of a glycoside hydrolase family 5 β-mannanase), a family 27 carbohydrate-binding module of a *Thermotoga maritima* β-mannanase (TmCBM27) is fused to its C-terminus linked with a flexible peptide F (GGGGS)_3_ and rigid peptide R (EAAAK)_3_ (Li et al., 2017a). Thus two fusion β-mannanases, AuMan5A-R-M and AuMan5A-F-M are designed and their encoding genes are constructed, and expressed along with the parental enzyme in *Pichia pastoris* GS115. Among the three recombinant β-mannanases (reAuMan5A, reAuMan5A-R-M, and reAuMan5A-FM), the optimum temperature of reAuMan5A-R-M is similar to the parental enzyme reAuMan5A but its melting temperature (T_m_) and thermostability are 8.4 and 8.0°C higher than the wild type enzyme. Moreover, the *K*_m_ values of reAuMan5A-R-M toward Konjac gum, guar gum and Locust bean gum increase significantly and its catalytic efficiency values (*K_cat_/K_m_*) decrease significantly in comparison to reAuMan5A. The superior thermostability and substrate affinity of reAuMan5A-R-M make it a suitable candidate for many industrial processes.

Numerous studies have shown that the alteration of the loop structures in proximity to the active centers of the glycoside hydrolyzes may enhance their catalytic efficiency and/or thermostability. Dong et al. (2016) have sought to improve the characteristics of an *Aspergillus usamii* derived β-mannanase (AuMan5A) by substitution of its loop structure. Based on enzymatic characteristics and structural analysis of various GH 5 β-mannanases, three mutants (AuMan5A, AuMAN5A-An, AuMAN5A-AF) are designed by substituting a piece of loop structure with seven amino acids in the substrate binding groove of AuMan5A with the corresponding sequences of three other family 5 β-mannanases, respectively. AuMan5A and its mutants are expressed in *Pichia pastoris* and characterized. The expressed recombinant AuMan5A-Af (re-AuMan5A-Af) is the best performing mutant. It shows a T_m_ value of 76.6°C, temperature optimum of 75°C and a half-life of 480 min at 70°C which are 12.1 and 10°C higher and 48-fold longer than those of the parental enzymes, respectively.

A β-mannanase (MAN) derived from *B. subtilis* B10-02 and over expressed in *B. subtilis* 168 is unstable under acidic conditions, thus restricting its use in food and feed industry (Xu et al., 2013). To enhance the acid stability of BsMAN6H several surface exposed basic amino acid residues are altered to neutral or acidic ones by site-directed mutagenesis. Among the variants produced, the variant H54D shows a decrease in the pH optima from 6.5 to 5.5 and an increased acid stability in the pH range of 4.0–5.5. In addition, the mutant H54D shows an enhanced enzyme activity (of 3207.82 U/ml) compared to parental enzyme. The enhanced enzymatic activity in H54D is attributed to, greater number of hydrogen bonds set up between the Asp54 and the neighboring amino acid residues, and negative potential around the mutated site which significantly altered the modeled electrostatic potential on the protein surface.

### Higher Protease Resistance of β-Mannanase

Modification through artificial glycosylation is a technology that can improve the characteristics of a protein by changing its structure with a carbohydrate chain. Hu et al. (2017) employ rationally designed N-glycosylation to enhance the protease resistance and stability of a recombinant β-mannanase (MAN 47) from *Armillariella tabescens.* To facilitate N-glycosylation an enhanced aromatic sequon sequence was introduced into specific MAN47 loop regions. The mutant MAN47 enzymes (g-123 and g-347) are expressed in yeast, glycosylated as expected and characterized. The pH stability, thermostability and protease resistance improved significantly when compared to the wild type enzyme. It is speculated that the carbohydrate chain protects protease target sites, interacts with amino acid residues and enhances the hydrophilicity of β-mannanase. Thus, as a result the molecular rigidity of β-mannanase is increased. Thereby, mutants of β-mannanase MAN47 with improved stability are obtained. An enzyme with multiple improved stability characteristics has broad applications in different industrial fields especially in the detergent and animal feed industries.

Similarly, in another study protease resistance of β-mannanase is improved to make it suitable for the feed industry which is necessary because of the presence of secretory proteases in the digestive tract. Li et al. (2013) employ rational method for improvement in trypsin resistance of β-mannanase. By using computational design via H-bond analysis and molecular dynamics simulations, optimal mutations of K280N, K371Q, and K280N/K107H/R102N are predicted. The trypsin resistance of mutants produced (K371Q, K280N/K107H/R102N, K280N) and the wild type β-mannanase is determined. There is a significant increase in trypsin resistance of triple mutant compared to wild type showing that molecular rational evolution can be used to increase the trypsin resistance of an enzyme.

## Future Prospects

The application of β-mannanases in commercial sector has been steadily increasing over the past few decades. The cut-throat competition forces the enzyme industry to continuously look for better and newer enzymes. Researchers and engineers are looking for novel microbial β-mannanases that fulfill the requirements of industry. In general, conventional methods for β-mannanase discovery are expensive, have low success rate and time consuming. Metagenomics is an excellent alternative approach to these methods and can help in discovering novel β-mannanases from mother nature. However, β-mannanases from natural sources are sometimes not suitable for rigorous industrial biocatalytic processes because either the lower yield of the enzyme or other important catalytic properties. Thus, tailoring or bioengineering of the enzyme is required.

Advances in protein engineering has brought forth new opportunities to introduce predesigned changes to create customized β-mannanases having the desirable properties. However, in engineering of β-mannanases a few challenges lie ahead: (1) It must be considered that enhancing the thermostability of an enzyme often decreases its overall flexibility, therefore the produced enzyme will likely have lower catalytic efficiency; (2) Before the engineered β-mannanases can be used in food and feed sector, there’s need to investigate how these designed proteins interact with biological systems; (3) The paucity of general rules in prioritization of enzymatic properties must be improved while selection of appropriate methods is also a difficult issue in bioengineering of β-mannanase. Collection of successful studies in β-mannanase engineering should provide suitable guidelines; (4) The quest for an ideal β-mannanase is ongoing even after many research efforts, while the *de novo* design of highly optimized industrial β-mannanase remains still elusive. There is need to enhance the mechanistic knowledge of the structure-function and dynamics relationships, so as to improve the algorithm for computational enzyme design. Once these advanced technologies are developed, designed β-mannanases will be easier to manufacture and industrialize.

Finally, a principal obstacle in the marketing of enzymatic processes is the cheap, large scale production of β-mannanase enzyme. To realize this goal, strategies need to be discovered which help facilitate the cheap production of bulk β-mannanase. It is hoped that in the near future, newer methods for simple and affordable production of β-mannanase, which can effectively meet the requirements of different industries will be discovered.

### Conclusion

β-mannanases have been used in a wide range of industries such as feed, detergent, biorefinery and textile. The production and use of β-mannanases are on the rise due to increased awareness of their utility and the incorporation of enzyme engineering and gene manipulation techniques. Now, there is an urgent need to create β-mannanases better suited to demands of the industrial sector at cheap costs, so that the use of noxious chemicals in the industrial sector is replaced by eco-friendly biocatalysts. The state and the higherups should take charge of encouraging this change so that industrial products are produced more cleanly and the risk of eco-pollution is reduced.

## Author Contributions

AD wrote the manuscript. KM reviewed the manuscript and made the necessary corrections. Both authors contributed to the article and approved the submitted version.

## Conflict of Interest

The authors declare that the research was conducted in the absence of any commercial or financial relationships that could be construed as a potential conflict of interest.
